# MSC-AS1 knockdown inhibits cell growth and temozolomide resistance by regulating miR-373-3p/CPEB4 axis in glioma through PI3K/Akt pathway

**DOI:** 10.1007/s11010-020-03937-x

**Published:** 2020-10-26

**Authors:** Chong Li, Shiyu Feng, Ling Chen

**Affiliations:** Department of Neurosurgery, Chinese PLA General Hospital, No. 28, Fuxing Rd, Haidian District, Beijing, 100853 China

**Keywords:** Glioma, Temozolomide, MSC-AS1, miR-373-3p, CPEB4, PI3K/Akt

## Abstract

Long non-coding RNAs (lncRNAs) have been widely reported to regulate the development and chemoresistance of a variety of tumors. Temozolomide (TMZ) is a first-line chemotherapy for treatment of glioma. However, the effect and the regulatory mechanism of lncRNA MSC-AS1 (MSC-AS1) in TMZ-resistant glioma remain unrevealed. Levels of MSC-AS1, microRNA-373-3p (miR-373-3p), and cytoplasmic polyadenylation element binding protein 4 (CPEB4) were determined by quantitative real-time polymerase chain reaction (qRT-PCR). All protein expression was detected by western blot. Cell viability and the half maximal inhibitory concentration (IC_50_) value of TMZ was assessed by cell counting kit-8 (CCK-8) assay. Cell cloning ability and apoptosis were examined by colony formation and flow cytometry assays, respectively. Dual-luciferase reporter and RNA immunoprecipitation (RIP) assays were performed to verify the correlation between miR-373-3p and MSC-AS1 or CPEB4. The xenograft models were established to determine the effect of MSC-AS1 in vivo. MSC-AS1 was up-regulated in TMZ-resistant glioma tissues and cells, and glioma patients with high MSC-AS1 expression tend to have lower overall survival rate. MSC-AS1 suppression reduced the IC_50_ value of TMZ and proliferation, promoted apoptosis and TMZ sensitivity, and affected PI3K/Akt pathway in TMZ-resistant glioma cells. MSC-AS1 acted as miR-373-3p sponge, and miR-373-3p directly targeted CPEB4. Silencing miR-373-3p reversed the promoting effect of MSC-AS1 or CPEB4 knockdown on TMZ sensitivity. Furthermore, MSC-AS1 knockdown inhibited TMZ-resistant glioma growth in vivo by regulating miR-373-3p/CPEB4 axis through PI3K/Akt pathway. Collectively, MSC-AS1 knockdown suppressed cell growth and the chemoresistance of glioma cells to TMZ by regulating miR-373-3p/CPEB4 axis in vitro and in vivo through activating PI3K/Akt pathway.

**Electronic supplementary material:**

The online version of this article (10.1007/s11010-020-03937-x) contains supplementary material, which is available to authorized users.

## Introduction

Glioma is one of the most common malignant brain tumors, accounting for 40–50% of intracranial tumors [1]. Despite improvements in the clinical therapeutic approaches for glioma, the median survival time of patients is still less than 1 year [2]. Temozolomide (TMZ) is a kind of alkylation anti-tumor drug, and it is widely employed as the first-line chemotherapy in glioma patients [3, 4]. But, recent studies indicated that patients developed resistance to TMZ treatment, resulting in unsatisfactory treatment effect of TMZ [5]. So it is urgently needed to figure out the mechanisms of TMZ resistance so as to improve the effect of TMZ chemotherapy.

Recently, numerous studies have reported that dysregulation of non-coding RNAs (ncRNA), including long non-coding RNAs (lncRNAs) and microRNAs (miRNAs), plays an important role in tumors development [6, 7]. In general, lncRNAs can act as competing endogenous RNAs (ceRNAs) of miRNAs to promote or inhibit tumor tumorigenesis and drug resistance [8, 9]. For instance, lncRNA CASC2 restrained the growth and TMZ resistance of glioma cells by sponging miR-181a [10]. lncRNA XIST could enhance the resistance of glioma cells to TMZ through interacting with miR-29c [11]. lncRNA MSC-AS1 (MSC-AS1) has been found to be up-regulated in kidney renal clear cell carcinoma [12] and pancreatic ductal adenocarcinoma [13] and plays a carcinogenic role. More than that, MSC-AS1 could facilitate osteogenic differentiation and attenuate osteoporosis via binding to miR-140-5p [14]. However, the expression pattern and mechanism of MSC-AS1 in glioma remain unknown, let alone the regulation of TMZ resistance. miR-373 was reported to be decreased in glioma, and the overall survival was poorer in glioma patients with low miR-373 expression [15]. Interestingly, miR-373-3p was boosted in tongue squamous cell carcinoma and esophageal squamous cell carcinoma, and promoted tumor cell development [16, 17], suggesting that miR-373-3p has a dual role. Besides, miR-373-3p was degraded in pancreatic carcinoma, and repressed cell growth, metastasis, and gemcitabine resistance [18]. Thus, we wondered whether miR-373-3p could modulate the TMZ resistance in glioma cells.

Cytoplasmic polyadenylation element binding protein 4 (CPEB4), which is a member of the CPEB family, has been shown to be aggrandized in many malignant tumors, such as gastric cancer [19] and breast cancer [20]. Many reports have also revealed the role of CPEB4 in glioma, as it was up-regulated in glioma, and it could be regulated by the lncRNA FOXD2-AS1/miR-98-5p axis to participate in glioma cell proliferation, metastasis, and TMZ resistance [21]. However, whether there was a link between miR-373-3p and CPEB4 in TMZ-resistant glioma remains unclear. In addition, previous study has shown that inhibition of PI3K/AKT pathway affected the function of CPEB4 [22]. Therefore, whether MSC-AS1 activates the PI3K/AKT signaling pathway in TMZ-resistant glioma remains to be further elaborated.

In this research, MSC-AS1 expression in TMZ-resistant glioma tissues and cells, and the impact of MSC-AS1 on proliferation, apoptosis, PI3K/AKT pathway, and TMZ resistance in TMZ-resistant glioma cells were investigated. Furthermore, we also explored the potential molecular mechanisms mediated by MSC-AS1.

## Materials and methods

### Clinical specimens and cell culture

50 Glioma tissues and 25 peritumoral brain edema tissues (normal tissues) were collected at Chinese PLA General Hospital. The sample tissues were collected with glioma patient consent and all participants signed informed consent forms. The detailed information of patients is provided in Table 1. Glioma patients were divided into resistant group (*n* = 25) and sensitive group (*n* = 25) based on their sensitivity to TMZ treatment. Tissues were frozen in liquid nitrogen after collection. The study was approved by the Ethics Committee of Chinese PLA General Hospital.

**Table 1:** The correlation of MAS-AS1 expression and the clinical characteristic of Glioma patients

Characteristics		MAS-AS1 expression		*P*-value
		High (*n*, %)	Low (*n*, %)	
Age (years)				
< 60	20	12	8	0.3868
≥ 60	30	13	17	
Gender				
Male	32	18	14	0.3772
Female	18	7	11	
Tumor size (cm)				
< 3	21	5	16	0.0037
≥ 3	29	20	9	
WHO classification				
I + II	35	13	22	0.0121
III + IV	15	12	3	
Lymph node metastasis				
Yes	26	9	17	0.0465
No	24	16	8	
TMZ drug resistance				
TMZ sensitive	25	7	18	0.0042
TMZ resistant	25	18	7	

Human glioma cell lines LN229 and SHG-44 and renal epithelial cell line 293T were obtained from Procell (Wuhan, China), cultured in Roswell Park Memorial Institute 1640 (RPMI 1640, HyClone, South Logan, UT, USA) supplemented with 10% fetal bovine serum (FBS, Gibco, USA) at 37 °C with 5% CO_2_. TMZ-resistant cell lines were established as described by Tian et al. [23]. Briefly, LN229 and SHG-44 cells were treated with an initial TMZ concentration of 1 μM for 2 weeks. Then, the TMZ dose was doubled, and each dose was hatched for 2 weeks, and the final concentration was added to 400 μM. The established TMZ-resistant cells were named as LN229/TR and SHG-44/TR.

### Transfection

Small interfering RNA against MSC-AS1 1, 2, and 3 (si-MSC-AS1-1, si-MSC-AS1-2, si-MSC-AS1-3) or CPEB4 (si-CPEB4) and control (si-NC), miR-373-3p mimic, inhibitor, and their matched controls (mimic NC, inhibitor NC) were synthesized by RiboBio (Guangzhou, China). Lentivirus packaged sh-MSC-AS1 plasmid (RiboBio) was used to suppress the expression of MSC-AS1, and sh-NC acted as the negative control. These structures were transfected into cells by Lipofectamine 3000 (Invitrogen, Carlsbad, CA, USA).

### Quantitative real-time polymerase chain reaction (qRT-PCR)

Glioma tissues and cells were collected, and their total RNAs were extracted by TRIzol (Invitrogen). The reverse transcription was performed using a Reverse Transcription Kit (Takara, Wuhan, China). The relative expression was detected by qRT-PCR using SYBR Green PCR Master Mix (Takara) on the ABI 7500 Fast Real-Time PCR system (Applied Biosystems, Foster City, CA, USA). U6 and glyceraldehyde-3-phosphate dehydrogenase (GAPDH) were used as endogenous controls for miR-373-3p and MSC-AS1 or CPEB4. The primers explored were listed as below: MSC-AS1, Forward (F): 5′-GCCAGTCAGAAAATGAGGAAC-3′ and Reverse (R): 5′-CCAGTTGGGTGAACAGGAC-3′; miR-373-3p, F: 5′-GAAGTGCTTCGATTTTGCC-3′ and R: 5′-GAATACCTCGGACCCTGC-3′; CPEB4, F: 5′-TGGGGATCAGCCTCTTCATA-3′ and R: 5′-CAATCCGCCTACAAACACCT-3′; GAPDH, F: 5′-GCACCGTCAAGGCTGAGAAC-3′ and R: 5′-ATGGTGGTGAAGACGC CAGT-3′; and U6, F: 5′-CTCGCTTCGGCAGCACA-3′ and R: 5′-AACGCTTCACGAATTTGCGT-3′.

### Western blot assay

The proteins from glioma tissues and cells were lysed in RIPA buffer (Thermo Fisher Scientific, Waltham, MA, USA), separated using sodium dodecyl sulfate polyacrylamide gel electrophoresis (SDS-PAGE), and then transferred to polyvinylidene fluoride membranes (PVDF, Invitrogen). After blocked with 5% skim milk powder for 2 h, the membranes were probed with primary antibodies overnight at 4 °C and maintained in horseradish peroxidase-linked secondary antibodies (1:4000, Abcam, Cambridge, MA, USA) for 1 h. Finally, the proteins were visualized by chemiluminescence and the relative protein expression was analyzed by the ImageJ software and normalized to GAPDH. The primary antibodies were myeloid cell leukemia-1 (MCL-1, 1:1000, Abcam), multidrug resistance-associated protein-1 (MRP-1, 1:500, Abcam), cyclinD1 (1:2000, Abcam), B-cell lymphoma-2-associated x (Bax, 1:2000, Abcam), caspase-3 (1:2000, Abcam), CPEB4 (1:1000, Thermo Fisher Scientific), PI3K (1:1000, Abcam), P-PI3K (1:1000, Abcam), AKT (1:1000, Abcam), P-AKT (phospho Ser473) (1:1000, Abcam), and GAPDH (1:2000, Abcam).

### TMZ chemosensitivity and cell viability assay

LN229/TR and SHG-44/TR cells after stable transfection were tiled into the 96-well plates and treated with different concentrations of TMZ (0–3 μM) for 48 h. The concentration selection was based on the study of Tan et al. [24]. Then, 10 μL cell counting kit-8 (CCK-8, Dojindo, Shanghai, China) was added to the cells and incubated for another 3 h. The absorbance at 450 nm was measured by a Microplate reader. The half maximal inhibitory concentration (IC_50_) value of TMZ was employed to assess the sensitivity of TMZ in glioma cells.

### Colony formation assay

LN229/TR and SHG-44/TR cells were inoculated at the density of 800 cells/well in 6-well plates and transfected. Cells were cultured in RPMI 1640 with 10% FBS and 300 μmol/L TMZ. 2 weeks later, the supernatant of medium was discarded and cells were fixed and stained with 0.1% crystal violet (Beyotime, Beijing, China) for 20 min. The stained and visible colonies were counted and photographed.

### Cell apoptosis analysis

Cell apoptosis rate was determined by using an Annexin V-fluorescein isothiocyanate (FITC)/propidium iodide (PI) apoptosis detection kit (Keygen, Beijing, China). TMZ-resistant glioma cells were first transfected. After 48 h later, cells were harvested and washed. Then, cells were stained with 5 μL Annexin V-FITC and 5 μL PI for 15 min in the dark. Cell apoptosis was analyzed by flow cytometry (BD Biosciences, Franklin Lake, NJ, USA).

### Dual-luciferase reporter assay

Bioinformatics analysis (starBase v3.0) was used to predict targets of miR-373-3p. To verify the potential interaction between miR-373-3p and MSC-AS1 or CPEB4, we subcloned MSC-AS1 or CPEB4 with site-directed mutation or not in the miR-373-3p binding site into the pmirGLO vector (Promega, Fitchburg, WI, USA). Therefore, luciferase activity represents the expression and activity of miR-373-5p. The products were named as MSC-AS1 wt, MSC-AS1 mut, CPEB4 3′UTR wt, and CPEB4 3′UTR mut, and they were co-transfected into HEK-293T cells with miR-373-3p mimic or mimic NC, respectively. 24 h later, the cells were lysed and the luciferase activity was examined by a dual-luciferase reporter assay kit (Promega).

### RNA immunoprecipitation (RIP) assay

EZMagna RIP kit (Millipore, Billerica, MA, USA) was used in RIP assay. LN229/TR and SHG-44/TR cells were collected and cleaved by cell lysis, following incubated with magnetic beads conjugated to Argonaute-2 (Ago2) antibody or immunoglobulin G (IgG) antibody. Subsequently, the RNAs from the beads were purified and extracted by TRIzol. The levels of MSC-AS1 and miR-373-3p were measured by qRT-PCR.

### Tumor formation assay

LN229/TR and SHG-44/TR cells transfected with sh-MSC-AS1 or sh-NC were subcutaneously injected into 4-week-old male nude mice (*n* = 6 each group). At 10 days after the injection, tumor volume was measured and then detected every other 4 days. TMZ (25 mg/kg) was intraperitoneally injected into the mice every day when the tumors reached 100 mm^3^. The volume of the tumor was calculated by following formula: volume (mm^3^) = (longest tumor diameter × shortest tumor diameter^2^)/2. 30 days later, all mice were euthanized and tumor weight was measured. In addition, tumor tissues were stripped to extract the RNAs and proteins. The animal experiments were approved by the Animal Care and Use Committee of Chinese PLA General Hospital.

### Statistical analysis

Data were acquired with at least three replicates and shown as the mean ± standard deviation (SD). Comparisons among multiple groups were evaluated by one-way analysis of variance (ANOVA) and the differences between two groups were analyzed by Student’s *t*-test through GraphPad Prism 7. Statistical significance was defined as the *P* value less than 0.05.

## Result

### MSC-AS1 was highly expressed in TMZ-resistant in glioma cells and tissues

**Fig. 1:**
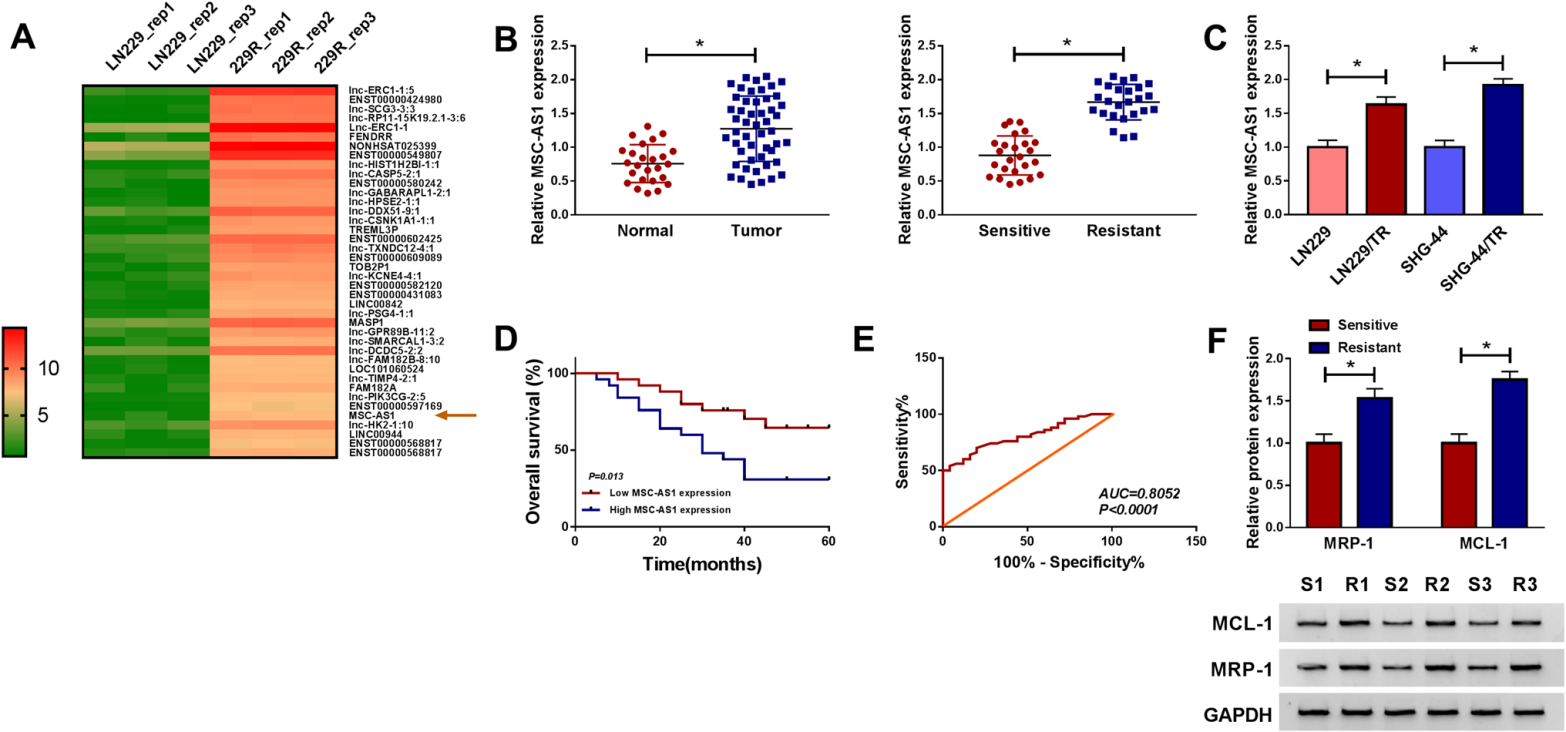
MSC-AS1 was highly expressed in TMZ-resistant in glioma cells and tissues. **a** 40 Up-regulated lncRNAs in TMZ-resistant glioma cell line (229R) compared to its parental cell line (LN299) were shown from downloaded the GSE113510 dataset. **b** MSC-AS1 expression in glioma and normal tissues, TMZ-resistant, and TMZ-sensitive glioma tissues was measured by qRT-PCR. **c** MSC-AS1 expression in LN229/TR and SHG-44/TR cells compared to their parental cells (LN229 and SHG-44) was analyzed by qRT-PCR. **d** The overall survival rate in glioma patients with high MSC-AS1 expression compared to patients with low MSC-AS1 expression was evaluated using Kaplan–Meier overall survival curve. **e** The diagnostic efficiency of MSC-AS1 in glioma. **f** The levels of MCL-1 and MRP-1 in TMZ-resistant and TMZ-sensitive glioma tissues were measured by western blot. *P < 0.05

To identify glioma resistance-related lncRNAs, we analyzed the expression profile of lncRNAs in the GSE113510 microarray dataset downloaded from the GEO database. As shown in Fig. 1a, 40 lncRNAs were up-regulated in TMZ-resistant glioma cells (229R). Excluding the miRNAs which its roles in cancers have not previously been reported, MSC-AS1 was the top up-regulated lncRNA. Research has shown that MSC-AS1 acts as a tumor promoter in hepatocellular carcinoma [25], nasopharyngeal carcinoma [26], and kidney renal clear cell carcinoma [12]. However, the effect of MSC-AS1 in glioma cancer is still limited. Thus, MSC-AS1 was selected for further research. We used qRT-PCR to validate MSC-AS1 expression in glioma tissues, and MSC-AS1 was significantly higher in glioma tissues than that normal tissues, and higher level of MSC-AS1 was observed in TMZ-resistant glioma tissues compared to TMZ-sensitive glioma tissues (Fig. 1b). Consistently, qRT-PCR results showed that MSC-AS1 expression was markedly increased in TMZ-resistant (TR) glioma cells (LN229/TR and SHG-44/TR) compared with their parental cells (LN229 and SHG-44) (Fig. 1c). Besides, glioma patients with high MSC-AS1 expression showed a shorter 5-year overall survival than that in glioma patients with low MSC-AS1 expression (Fig. 1d). And the receiver operating characteristic (ROC) analysis of the sensitivity and specificity showed that the area under the RPC curve (AUC) was 0.8052 for MSC-AS1 in detecting glioma patients from the healthy people (Fig. 1e).

MCL-1, an anti-apoptotic member of the BCL-2 family proteins, is frequently overexpressed in a variety of cancers [27]. A number of studies have confirmed the pivotal roles of MCL-1 in cancer cell survival and apoptosis resistance, as in vitro evidence documenting the pro-apoptotic and chemosensitization effects of Mcl-1 knockdown [28–30]. In TMZ-resistant glioma tissues, the expression levels of resistant marker proteins MCL-1 and MRP-1 were increased (Fig. 1f). To confirm that we have successfully generated the TMZ-resistant glioma cell lines, we also measured the expression of resistant marker proteins. As shown in Fig. S1a, the protein levels of MCL-1 and MRP-1 were distinctly facilitated in LN229/TR and SHG-44/TR cells in contrast to their parental cells. In addition, the viability of LN229/TR and SHG-44/TR cells was drastically enhanced in a TMZ (0–400 μM) dose-dependent manner, and the half maximal inhibitory concentration (IC_50_) value of TMZ in LN229/TR and SHG-44/TR cells was increased (Fig. S1b), suggesting TMZ-resistant glioma cell lines were successfully established. The results demonstrated that MSC-AS1 might play an important role in glioma resistance to TMZ.

### MSC-AS1 knockdown inhibited cell proliferation, induced TMZ sensitivity and cell apoptosis, and affected PI3K/AKT pathway in TMZ-resistant glioma cells

**Fig. 2:**
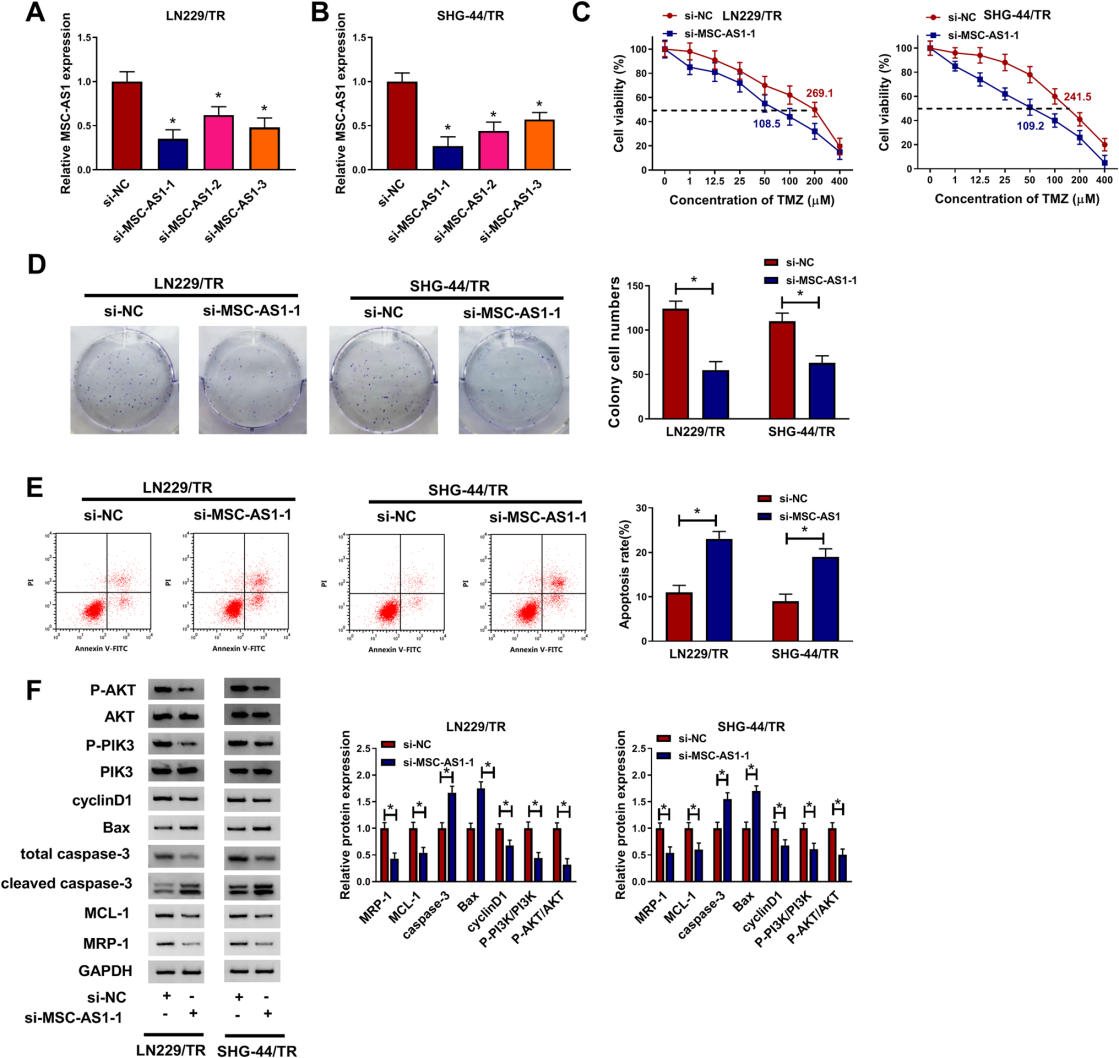
MSC-AS1 knockdown inhibited cell proliferation and induced TMZ sensitivity and cell apoptosis in TMZ-resistant glioma cells. LN229/TR and SHG-44/TR cells were transfected with si-NC or si-MSC-AS1-1. **a** and **b** MSC-AS1 expression was measured by qRT-PCR. **c** Cell viability and IC_50_ value of TMZ were evaluated using CCK-8 assay. **d** and **e** The number of cell colony and cell apoptosis rate were determined by colony formation assay and flow cytometry assay, respectively. **f** The protein levels of MCL-1, MRP-1, cyclinD1, Bax, P-PI3K, P-AKT, and caspase-3 were detected by western blot. *P < 0.05

Given that MSC-AS1 was highly expressed in TMZ-resistant glioma cell lines, we knocked down MSC-AS1 in LN229/TR and SHG-44/TR cells by transfection of si-MSC-AS1-1, si-MSC-AS1-2, and si-MSC-AS1-3. The efficiency of MSC-AS1 knockdown was determined by qRT-PCR. The data showed that MSC-AS1 expression was significantly decreased in LN229/TR and SHG-44/TR cells transfected with si-MSC-AS1-1, si-MSC-AS1-2, and si-MSC-AS1-3 compared to control group and the si-MSC-AS1-1 had the most obvious inhibitory effect (Fig. 2a, b). When MSC-AS1 was silenced in LN229/TR and SHG-44/TR cells, TMZ could decrease cell viability in a dose-dependent manner and the IC_50_ value of TMZ was reduced (Fig. 2c). Furthermore, colony formation assay showed that repression of MSC-AS1 degraded the rate of colony formation in LN229/TR and SHG-44/TR cells (Fig. 2d). On the contrary, flow cytometry assay demonstrated that interference with MSC-AS1 fortified the apoptosis rate of LN229/TR and SHG-44/TR cells (Fig. 2e). Meanwhile, western blot results indicated that the levels of MCL-1, MRP-1, and cell cycle-related protein cyclinD1 were down-regulated in LN229/TR and SHG-44/TR cells transfected with si-MSC-AS1-1 with respect to that cells transfected with si-NC, and the levels of pro-apoptotic proteins Bax and caspase-3 were up-regulated. In addition, the PI3K/Akt pathway-related proteins P-PI3K and P-AKT were markedly decreased (Fig. 2f). Moreover, we also utilized the si-MSC-AS1-3 to knockdown the expression of MSC-AS1. The data also showed that the cell colony was significantly decreased after silencing MSC-AS1 (Fig. 2a). These results suggested that silencing MSC-AS1 could suppress cell growth and TMZ resistance in TMZ-resistant glioma cells through PI3K/Akt pathway.

### MSC-AS1 directly targeted miR-373-3p

**Fig. 3:**
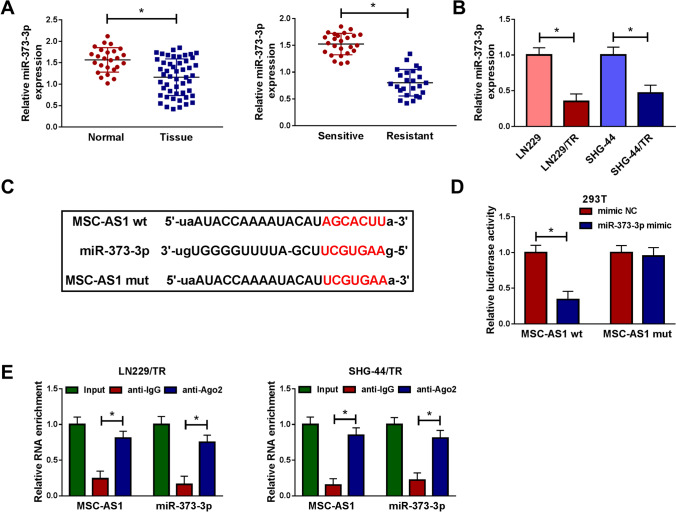
MSC-AS1 directly targeted miR-373-3p. **a** The relative expression of miR-373-3p in glioma and normal tissues, TMZ-resistant, and TMZ-sensitive glioma tissues was examined by qRT-PCR. **b** The relative expression of miR-373-3p in LN229/TR and SHG-44/TR cells compared to their parental cells (LN229 and SHG-44) was measured by qRT-PCR. **c** starBase v3.0 predicted there were binding sites between MSC-AS1 and miR-373-3p, and the mutant binding sites were displayed. **d** Dual-luciferase reporter assay was performed in 293T cells to verify the correlation between MSC-AS1 and miR-373-3p. **e** RIP assay was used to confirm MSC-AS1 could bind to miR-373-3p in LN229/TR and SHG-44/TR cells. *P < 0.05

Mounting evidence has revealed that lncRNAs act as molecular sponges and effectively inhibit miRNA function [8, 9]. Based on online lncRNA-miRNA binding databases (starBase v2.0), more than 50 miRNAs have the potential binding sites for MSC-AS1. We performed a systematic Literature search in pumped and screened 6 miRNAs (miR-29b-3p, miR-142-5p, miR-373-3p, miR-302a-3p, miR-524-3p, and miR-330-3p), which was down-regulated in glioma specimens. qRT-PCR assay was performed to detect the expression of these miRNAs in glioma specimens and TMZ-resistant glioma tissues. As shown in Fig. S3a, compared with normal tissues, miRNAs expression was noticeably decreased in glioma tissues. While in TMZ-resistant glioma tissues, the expression of miRNAs was further reduced compared to TMZ-resistant glioma tissues (Fig. S3b). Besides, the expression of miRNAs was also reduced in TMZ-resistant glioma cells LN229/TR (Fig. S3c) and SNG-44/TR (Fig. S3d). Among the top de-regulated lncRNAs was miR-373-3p. Considering miR-373 was decreased in glioma [15], and it could elevate the chemosensitivity of pancreatic carcinoma cells [18], we further investigated whether miR-373-3p was involved in glioma resistance to TMZ. We first measured miR-373-3p expression in glioma tissues by qRT-PCR. As presented in Fig. 3a, miR-373-3p expression was markedly reduced in glioma tissues and TMZ-resistant glioma tissues compared to normal tissues and TMZ-sensitive glioma tissues. Meanwhile, we observed that miR-373-3p expression was down-regulated in LN229/TR and SHG-44/TR cells versus their parental cells (LN229 and SHG-44) (Fig. 3b). Interestingly, miR-373-3p was predicted as a potential target gene for MSC-AS1 using starBase v3.0 online database (Fig. 3c). To verify whether miR-373-3p was a direct target gene of MSC-AS1, dual-luciferase reporter assay was performed in 293T cells. As shown in Fig. 3d, miR-373-3p mimic degraded the luciferase activity of MSC-AS1 wt, but not that of the MSC-AS1 mut, indicating a direct binding between them. RIP assay demonstrated that the enrichments of MSC-AS1 and miR-373-3p were enhanced in LN229/TR and SHG-44/TR cells combined with Ago2 antibody compared with the control group (Fig. 3e). These findings supported that MSC-AS1 could interact with miR-373-3p in TMZ-resistant glioma cells.

### Knockdown of miR-373-3p reversed the effects of MSC-AS1 depletion on TMZ-resistant glioma cells

**Fig. 4:**
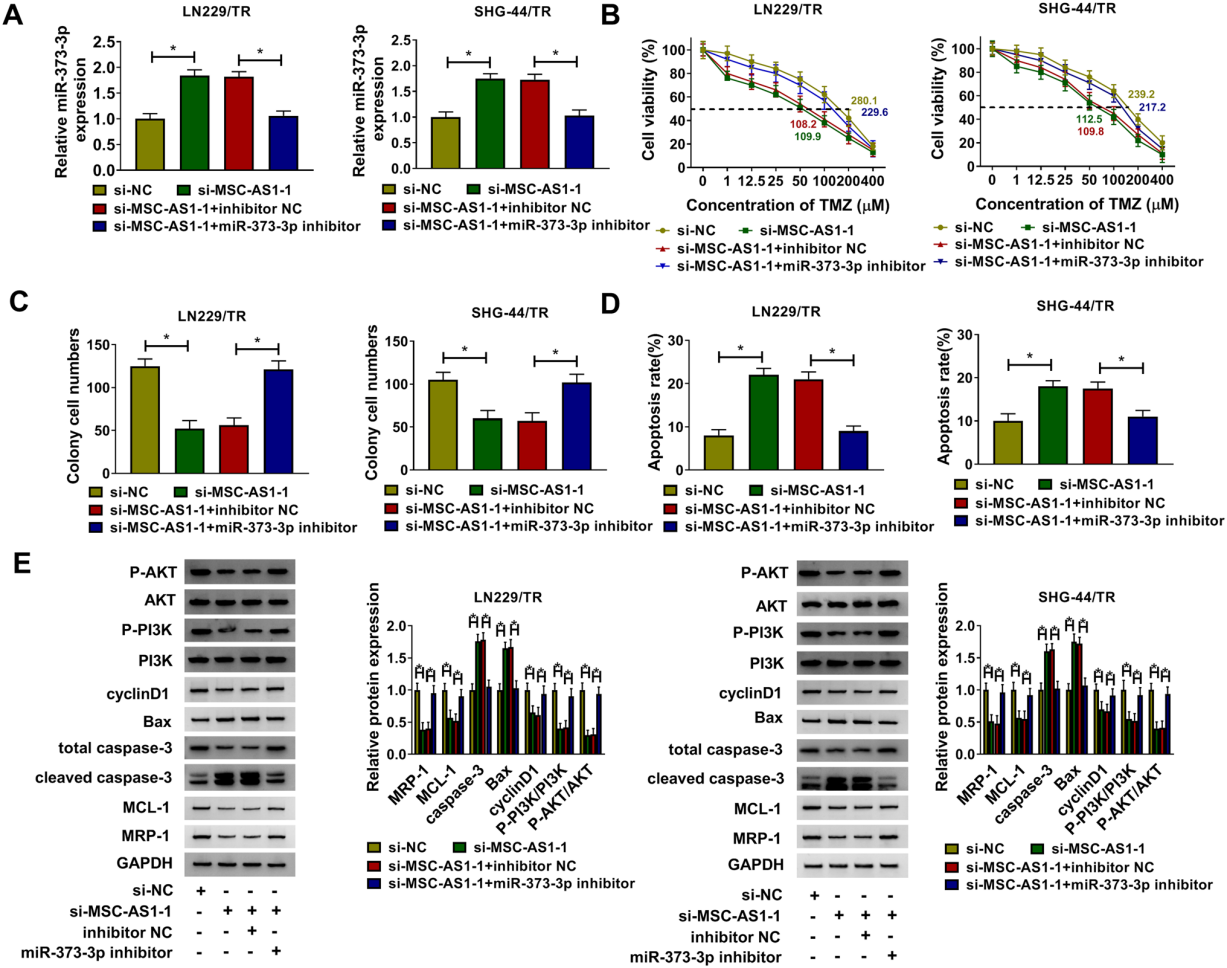
Knockdown of miR-373-3p reversed the effects of MSC-AS1 depletion on TMZ-resistant glioma cells. LN229/TR and SHG-44/TR cells were transfected with si-NC, si-MSC-AS1-1, si-MSC-AS1-1 + inhibitor NC, or si-MSC-AS1-1 + miR-373-3p inhibitor. **a** The relative expression of miR-373-3p was detected by qRT-PCR. **b** Cell viability and IC_50_ value of TMZ were estimated using CCK-8 assay. **c** and **d** The number of cell colony and cell apoptosis rate were assessed by colony formation assay and flow cytometry assay, respectively. **e** Western blot was performed to assess the levels of MCL-1, MRP-1, cyclinD1, Bax, P-PI3K, P-AKT, and caspase-3. *P < 0.05

We then evaluated whether MSC-AS1 regulated the proliferation, apoptosis, and TMZ resistance by targeting miR-373-3p in TMZ-resistant glioma cells. LN229/TR and SHG-44/TR cells were co-transfected with si-MSC-AS1-1 and miR-373-3p inhibitor. The results showed that miR-373-3p inhibitor could reverse the facilitated impact of si-MSC-AS1-1 on miR-373-3p expression (Fig. 4a). The cell viability and TMZ IC_50_ value (Fig. 4b) as well as the rate of colony formation (Fig. 4c) of LN229/TR and SHG-44/TR cells were obviously repressed by silencing MSC-AS1, whereas facilitated by interfering with miR-373-3p. The promoting effect of MSC-AS1 knockdown on TMZ-resistant glioma cells apoptosis could be counteracted by suppression of miR-373-3p (Fig. 4d). Meanwhile, MSC-AS1 knockdown decreased the levels of MCL-1, MRP-1, cyclinD1, P-PI3K, and P-AKT, and raised Bax and caspase-3 levels, whereas counteracted by inhibition of miR-373-3p (Fig. 4e). These results supported that MSC-AS1 knockdown inhibited cell proliferation and induced TMZ sensitivity and cell apoptosis in TMZ-resistant glioma cells by targeting miR-373-3p through regulating PI3K/Akt pathway.

### CPEB4 was a target of miR-373-3p

**Fig. 5:**
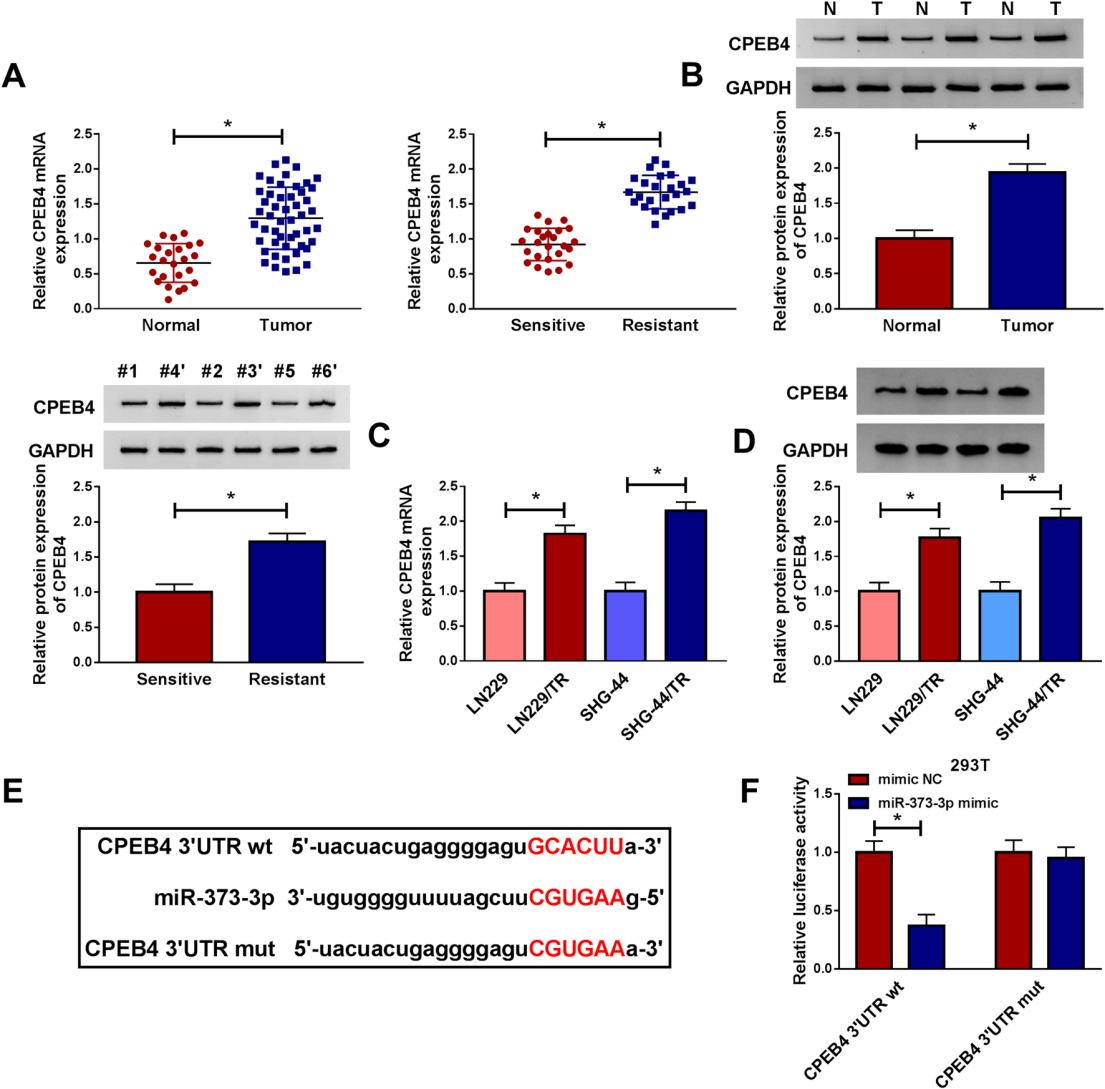
CPEB4 was a target of miR-373-3p. **a** and **b** The mRNA and protein levels of CPEB4 in glioma and normal tissues, TMZ-resistant, and TMZ-sensitive glioma tissues were analyzed by qRT-PCR and western blot, respectively. **c** and **d** The mRNA and protein levels of CPEB4 in LN229/TR and SHG-44/TR cells compared to their parental cells (LN229 and SHG-44) were evaluated by qRT-PCR and western blot, respectively. **e** starBase v3.0 predicted there were miR-373-3p binding sites in the 3′UTR of CPEB4. **f** Dual-luciferase reporter assay was used in 293T cells to verify the correlation between CPEB4 and miR-373-3p. *P < 0.05

In view of the key role of CPEB4 in glioma [21], we wondered whether there was a connection between CPEB4 and miR-373-3p. The expression of CPEB4 was first detected in glioma tissues. As shown in Fig. 5a, b, CPEB4 was up-regulated in glioma tissues and TMZ-resistant glioma tissues at mRNA and protein levels. Consistent with the results in tissues, the mRNA and protein levels of CPEB4 in LN229/TR and SHG-44/TR cells were notably enhanced than their parental cells (Fig. 5c, d). Since CPEB4 and miR-373-3p had opposite expression patterns, we then predicted the target genes of miR-373-3p by starBase v3.0 and found that there were complementary sites that bind to miR-373-3p in the 3′UTR of CPEB4 (Fig. 5e). Dual-luciferase assay revealed that the luciferase activity of CPEB4 3′UTR wt was degraded by miR-373-3p mimic, but there was no significant change in the luciferase activity of CPEB4 3′UTR mut in 293T cells (Fig. 5f), indicating CPEB4 was the target for miR-373-3p.

### Silencing miR-373-3p reversed the inhibitory effect of CPEB4 knockdown on TMZ resistance in TMZ-resistant glioma cells

**Fig. 6:**
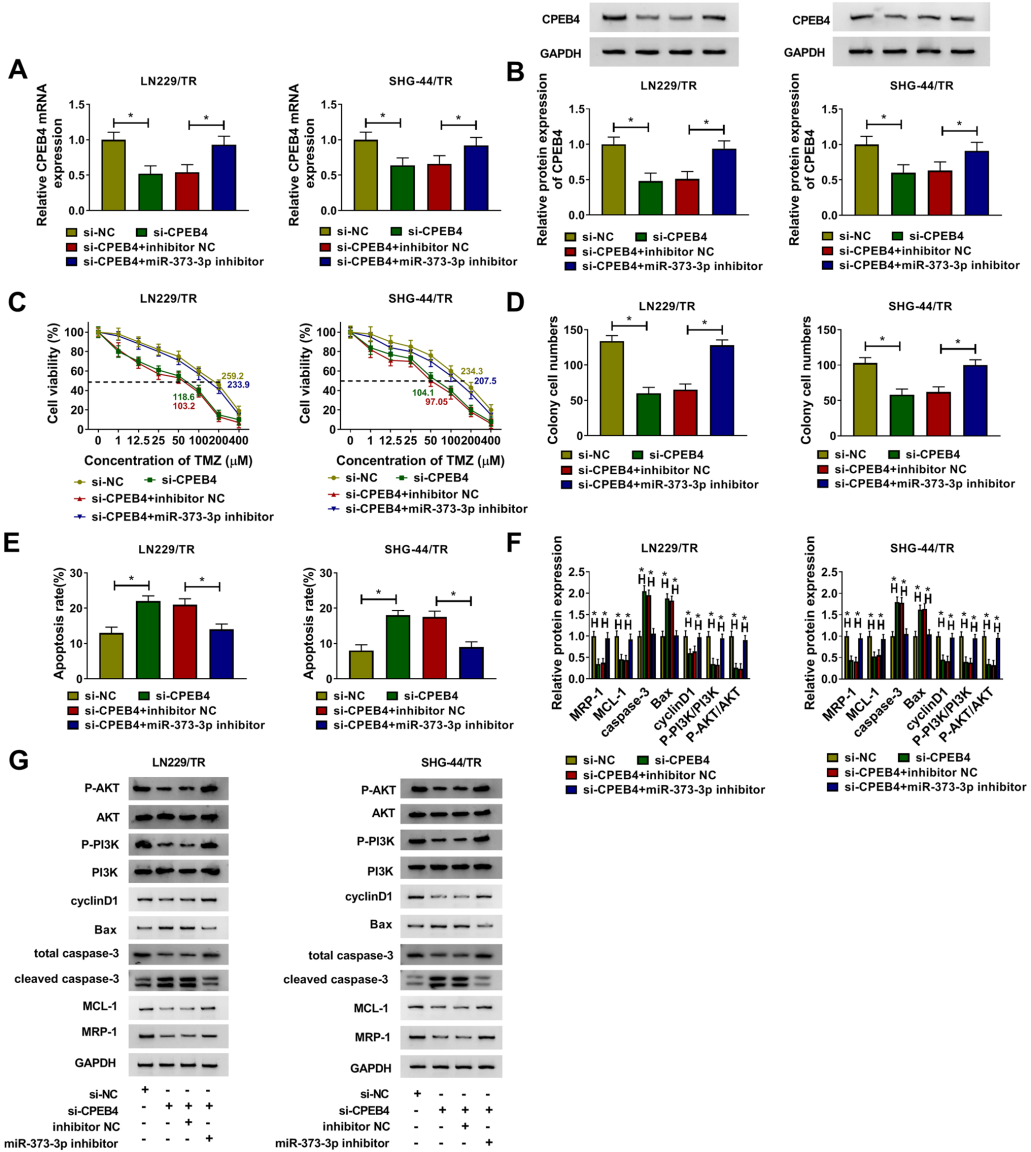
Silencing miR-373-3p reversed the inhibitory effect of CPEB4 knockdown on TMZ resistance in TMZ-resistant glioma cells. LN229/TR and SHG-44/TR cells were transfected with si-NC, si-CPEB4, si-CPEB4 + inhibitor NC, or si-CPEB4 + miR-373-3p inhibitor. **a** and **b** The mRNA and protein levels of CPEB4 were detected by qRT-PCR and western blot, respectively. **c** Cell viability and IC_50_ value of TMZ were examined by CCK-8 assay. **d** and **e** The number of cell colony and cell apoptosis rate were determined by colony formation assay and flow cytometry assay, respectively. **f** and **g** The levels of MCL-1, MRP-1, cyclinD1, Bax, and caspase-3 were measured by western blot. *P < 0.05

We further investigated the combined effect of miR-373-3p and CPEB4 on the proliferation, apoptosis, and TMZ resistance of TMZ-resistant glioma cells. As displayed in Fig. 6a, b, the mRNA level and protein level of CPEB4 were obviously down-regulated in glioma cells transfected with si-CPEB4, while this promoting effect was reversed in glioma cells co-transfected with si-CPEB4 and miR-373-3p. CCK-8 showed that the cell viability and IC_50_ value of TMZ were restrained by CPEB4 knockdown, whereas facilitated by miR-373-3p suppression in LN229/TR and SHG-44/TR cells (Fig. 6c). The inhibition of si-CPEB4 on the number of cell colony (Fig. 6d) and the promotion on cell apoptosis (Fig. 6e) could be reversed by miR-373-3p deficiency. Moreover, knockdown of CPEB4 degraded the levels of MCL-1, MRP-1, P-PI3K, P-AKT, and cyclinD1, and augmented the levels of Bax and caspase-3, whereas these effects were weakened by silencing miR-373-3p in LN229/TR and SHG-44/TR cells (Fig. 6f, g). These data suggested that CPEB4 could be targeted by miR-373-3p to regulate the proliferation, apoptosis, and TMZ resistance in TMZ-resistant glioma cells via activating PI3K/Akt pathway.

### MSC-AS1 knockdown could reduce the expression of CPEB4 by sponging miR-373-3p

**Fig. 7:**
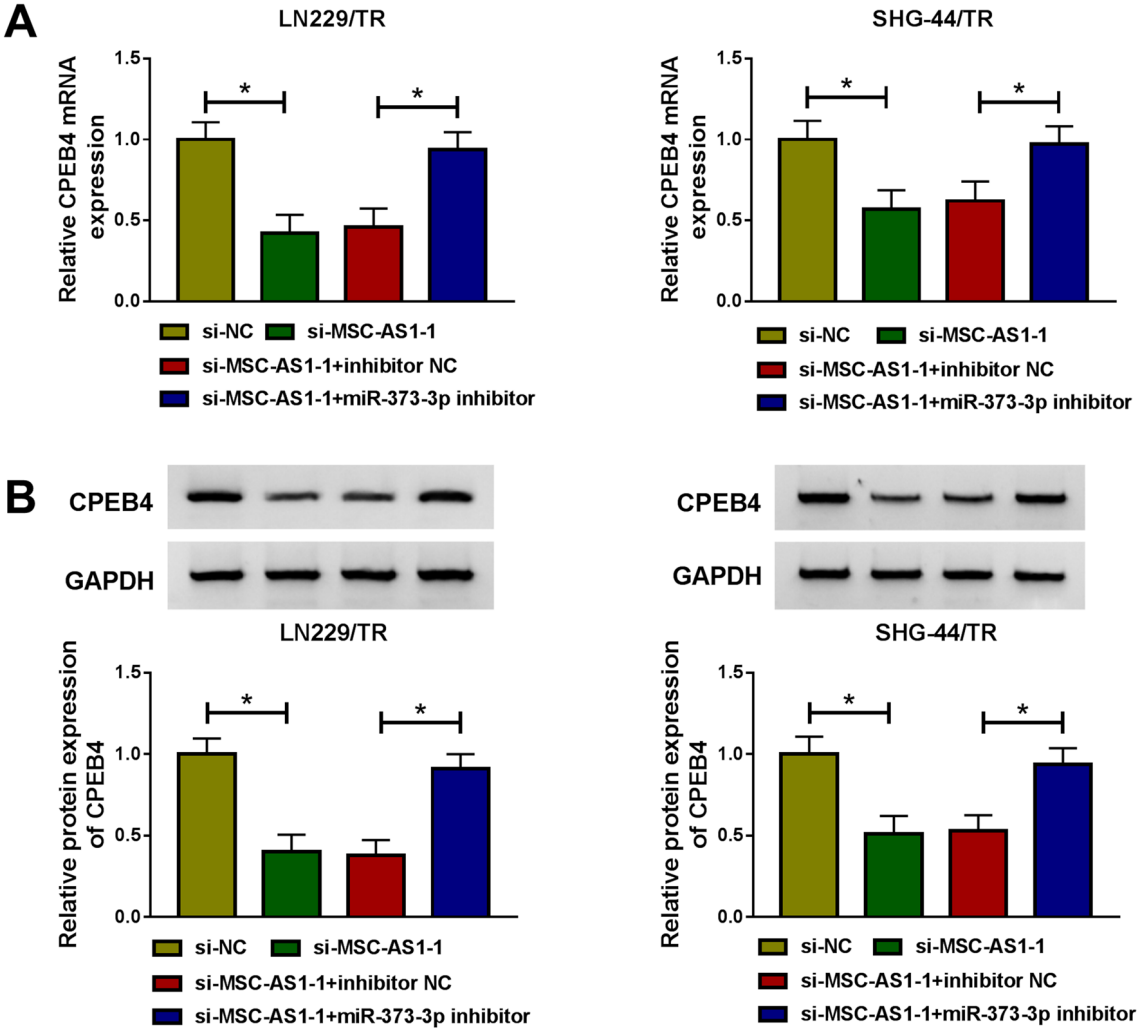
MSC-AS1 knockdown could reduce the expression of CPEB4 by sponging miR-373-3p. LN229/TR and SHG-44/TR cells were transfected with si-NC, si-MSC-AS1-1, si-MSC-AS1-1 + inhibitor NC, or si-MSC-AS1-1 + miR-373-3p inhibitor. **a** and **b** The mRNA and protein levels of CPEB4 were determined by qRT-PCR and western blot, respectively. *P < 0.05

Since MSC-AS1 could act as miR-373-3p sponge, and CPEB4 was the target for miR-373-3p, we further explored whether MSC-AS1 could regulate CPEB4 expression by targeting miR-373-3p. As shown in Fig. 7a, b, transfection with si-MSC-AS1-1 distinctly decreased the mRNA and protein levels of CPEB4. We also detected the CPEB4 expression was significantly down-regulated in si-MSC-AS1-3 group shown in Fig. S2b. However, the inhibitory effect of si-MSC-AS1-1 on CPEB4 expression was counteracted by miR-373-3p inhibitor in LN229/TR and SHG-44/TR cells, suggesting that MSC-AS1 could regulate CPEB4 expression by binding to miR-373-3p in TMZ-resistant glioma cells.

### Silencing MSC-AS1 suppressed the growth of TMZ-resistant glioma cells in vivo

**Fig. 8:**
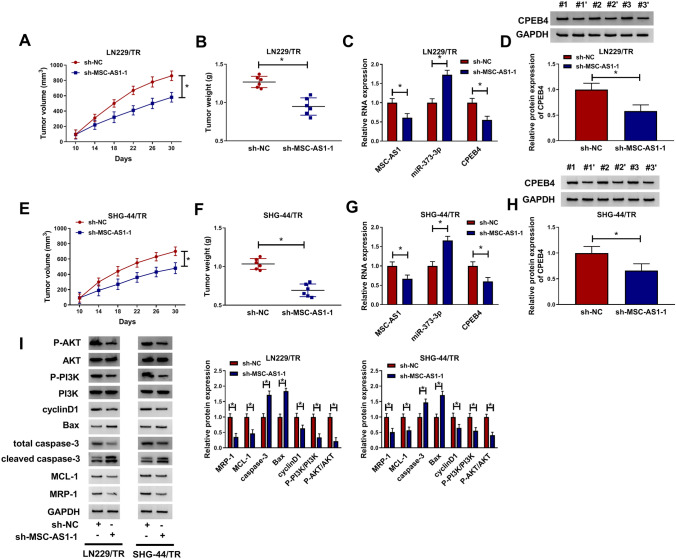
Silencing MSC-AS1 suppressed the growth of TMZ-resistant glioma cells in vivo. LN229/TR or SHG-44/TR cells transfected with sh-NC or sh-MSC-AS1 were inoculated into the flanks of nude mice. **a** and **b** Tumor volume and weight were measured in nude mice inoculated with LN229/TR cells. **c** The levels of MSC-AS1, miR-373-3p, and CPEB4 were detected by qRT-PCR in resected tumor tissues. **d** CPEB4 protein expression in resected tumor tissues was determined by western blot. **e** and **f** Tumor volume and weight were examined in nude mice inoculated with SHG-44/TR cells. **g** The levels of MSC-AS1, miR-373-3p, and CPEB4 in tumor tissues of nude mice inoculated with SHG-44/TR cells were analyzed by qRT-PCR. **h** The protein expression of CPEB4 was measured by western blot. **i** The protein levels of MCL-1, MRP-1, cyclinD1, Bax, P-PI3K, P-AKT, and caspase-3 in resected tumor tissues of nude mice inoculated with LN229/TR or SHG-44/TR cells were detected by western blot. *P < 0.05

To further explore the impact of MSC-AS1 knockdown on the growth of implanted LN229/TR tumors in vivo, LN229/TR cells stably transfected with sh-NC or sh-MSC-AS1 were inoculated into the flanks of nude mice. The results showed that MSC-AS1 knockdown inhibited tumor growth by reducing tumor volume and weight (Fig. 8a, b). qRT-PCR results indicated that lower levels of MSC-AS1 and CPEB4 were observed in tumors of LN229/TR-sh-MSC-AS1 group than that in the tumors of LN229/TR-sh-NC group, and higher level of miR-373-3p was shown in tumors of LN229/TR-sh-MSC-AS1 group (Fig. 8c), and the protein expression of CPEB4 was also decreased in LN229/TR-sh-MSC-AS1 group (Fig. 8d). Consistently, SHG-44/TR stably transfected with sh-NC or sh-MSC-AS1 was inoculated into the flanks of nude mice. Tumor volume and weight were reduced by MSC-AS1 inhibition (Fig. 8e, f). In addition, MSC-AS1 and CPEB4 levels were down-regulated and miR-373-3p was up-regulated in SHG-44/TR-sh-MSC-AS1 group (Fig. 8g); the protein expression of CPEB4 was also degraded in SHG-44/TR-sh-MSC-AS1 group (Fig. 8h). Additionally, compared to control group, the protein levels of MCL-1, MRP-1, P-PI3K, P-AKT, and cyclinD1 were dwindled in LN229/TR and SHG-44/TR-sh-MSC-AS1 groups, and the levels of Bax and caspase-3 were promoted (Fig. 8i). Therefore, MSC-AS1 knockdown impaired the growth of TMZ-resistant glioma cells in vivo by sponging miR-373-3p and hampering CPEB4 expression via PI3K/Akt pathway.

## Discussion

TMZ-based chemotherapy after surgical resection is a common clinical treatment for glioma patients [31]. However, the resistance of patients to TMZ makes the treatment effect unsatisfactory, so it is urgent to explore novel molecular resistance mechanism to improve the treatment strategy. In our study, we identified lncRNAs that might have prognostic value for glioma resistance through the GSE113510 microarray dataset, and finally identified MSC-AS1 as the study object.

MSC-AS1 was first reported to be augmented in hepatocellular carcinoma [32]. In addition, MSC-AS1 was an oncogenic gene of pancreatic cancer and kidney renal clear cell carcinoma, and MSC-AS1 overexpression hampered gemcitabine-induced apoptosis in pancreatic cancer cells [12, 13]. In this study, MSC-AS1 was not only up-regulated in glioma tissues but also increased in TMZ-resistant glioma tissues and cells. Furthermore, glioma patients with high MSC-AS1 expression had a poor 5-year overall survival. To understand whether MSC-AS1 was associated with TMZ resistance, we established the TMZ-resistant glioma cells, and the expression of drug-resistant marker proteins MCL-1 and MRP-1 was increased, and the cells showed higher cell viability in TMZ-resistant glioma cells, suggesting that the successful establishment of cell model. Subsequently, we found that MSC-AS1 knockdown impaired cell viability, proliferation, and the IC_50_ value of TMZ, and promoted apoptosis and sensitivity in TMZ-resistant glioma cells, indicating the potential role of MSC-AS1 in modulating the resistance of glioma cells to TMZ.

lncRNA has been shown to interact with miRNAs to regulate tumor cell progression, and MSC-AS1 was validated to modulate osteogenic differentiation and osteoporosis by sponging miRNA-140-5p [14]. We speculated whether MSC-AS1 can interact with other miRNAs to regulate TMZ resistance of glioma cells. As expected, down-regulated miR-373-3p in TMZ-resistant glioma was the downstream target miRNA of MSC-AS1. Previously, Zhou and Gao et al. revealed that lncRNA SNHG16 and HOXA-AS2 potentiated the tumorigenesis of glioma cells by decreasing miR-373 [33, 34], and miR-373-3p could inhibited proliferation and promoted apoptosis in gemcitabine-resistant pancreatic carcinoma cells [18]. In accordance with these data, when si-MSC-AS1-1 and miR-373-3p inhibitor were co-transfected into cells, the inhibitory effect of si-MSC-AS1-1 on TMZ resistance was reversed, suggesting that repression of MSC-AS1 degraded the TMZ resistance through increasing miR-373-3p in TMZ-resistant glioma cells.

It is well established that miRNA could regulate mRNA activity by binding to the 3′UTR of target gene [35]. CPEB4 was an oncogenic gene that was up-regulated in a variety of tumors [36], and it was considered a potential marker for defining metastatic cancers [37]. PI3K/Akt pathway was the CPEB4 downstream signaling pathway which regulated various tumor progression [38–40]. In the present study, CPEB4 was raised in TMZ-resistant glioma, and it could be targeted by miR-373-3p. Similarly, down-regulation of CPEB4 could inhibit cell proliferation and viability, elevate apoptosis and TMZ sensitivity, and affect the PI3K/Akt pathway-related proteins, whereas these effects were overturned by miR-373-3p inhibition. Our results were consistent with previous studies, in which Gu et al. showed that FOXD2-AS1 deficiency can hinder cell proliferation and TMZ resistance and promote apoptosis in TMZ-resistant glioma cells by up-regulating miR-98-5p and decreasing CPEB4 [41], and we revealed that MSC-AS1 knockdown could impede cell proliferation, viability, and TMZ resistance and facilitate apoptosis by increasing miR-373-3p and reducing CPEB4 in vitro and in vivo through activating PI3K/Akt pathway.

## Conclusion

In conclusion, MSC-AS1 suppression degraded CPEB4 expression by sponging miR-373-3p, thereby suppressing cell viability, IC_50_ value of TMZ, proliferation, TMZ resistance, and elevating apoptosis in TMZ-resistant glioma cells in vitro and in vivo via PI3K/Akt pathway, indicating that MSC-AS1 might be a prognostic marker in TMZ-based glioma chemotherapy.

## Electronic supplementary material

Below is the link to the electronic supplementary material.

Supplementary file1 (TIF 367 kb)

Supplementary file2 (TIF 148 kb)

Supplementary file3 (TIF 428 kb)
