# Supplementary figures and images for: MSC-AS1 knockdown inhibits cell growth and temozolomide resistance by regulating miR-373-3p/CPEB4 axis in glioma through PI3K/Akt pathway
Source: Mol Cell Biochem. 2020 Oct 26;476(2):699–713. doi: 10.1007/s11010-020-03937-x (PMC7873112; doi:10.1007/s11010-020-03937-x)

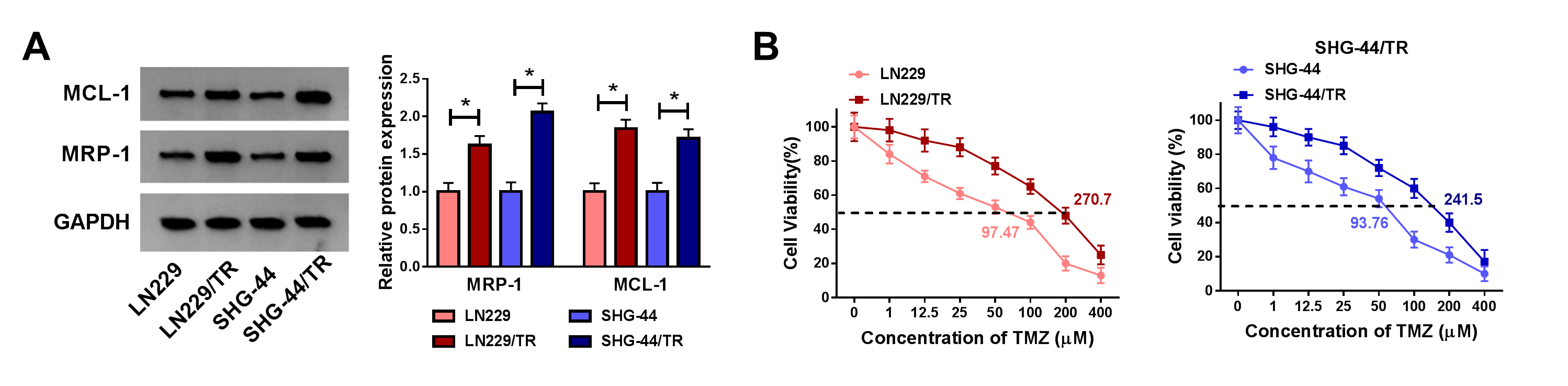

Supplement: Supplementary file 1 — Supplementary file1 (TIF 367 kb) [file 11010_2020_3937_MOESM1_ESM.tif]

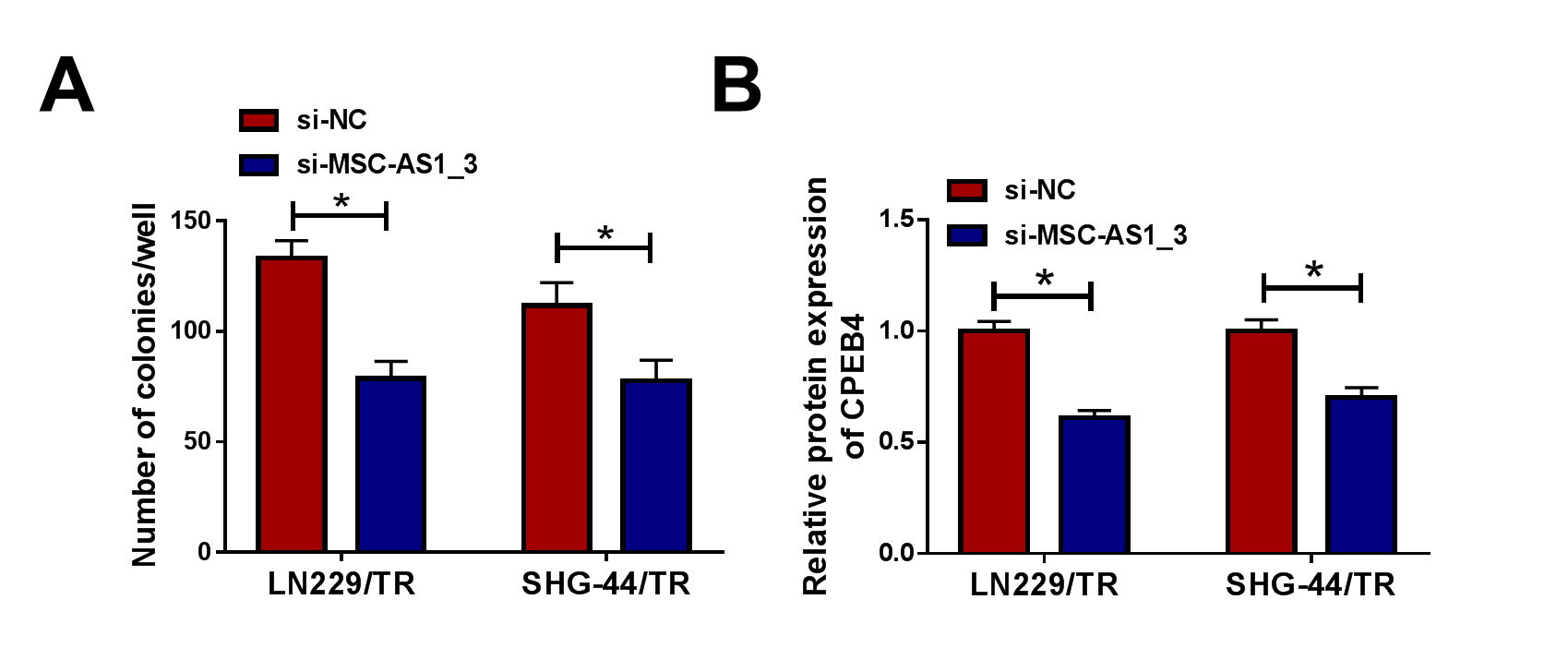

Supplement: Supplementary file 2 — Supplementary file2 (TIF 148 kb) [file 11010_2020_3937_MOESM2_ESM.tif]

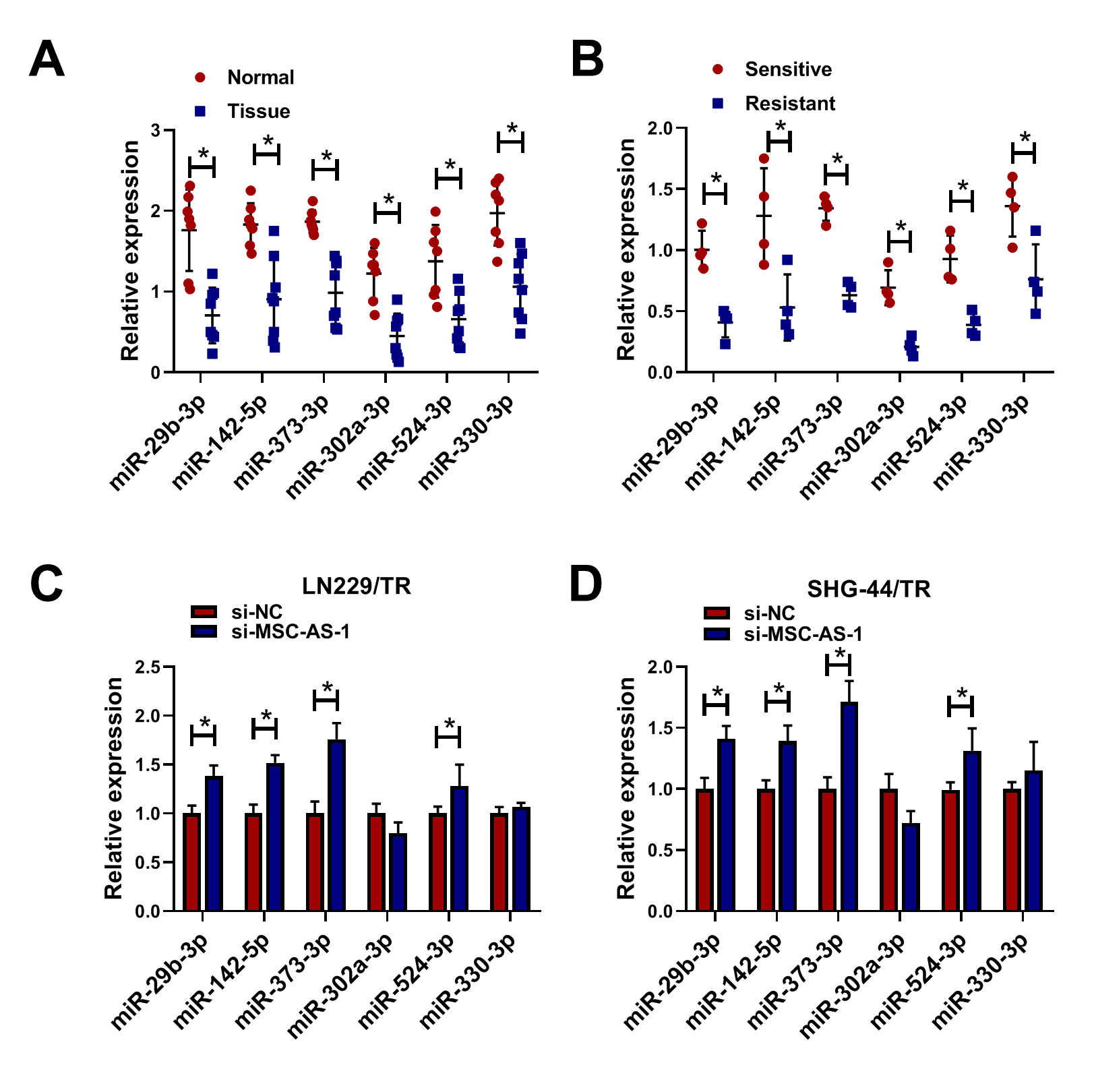

Supplement: Supplementary file 3 — Supplementary file3 (TIF 428 kb) [file 11010_2020_3937_MOESM3_ESM.tif]
